# Combined Airway and Bariatric Surgery (CABS) for Obstructive Sleep Apnea Patients with Morbid Obesity: A Comprehensive Alternative Preliminary Study

**DOI:** 10.3390/jcm11237078

**Published:** 2022-11-29

**Authors:** Chia-Chen Lin, Keng-Hao Liu, Li-Ang Lee, Li-Pang Chuang, Yu-Sheng Lin, Li-Jen Hsin, Wan-Ni Lin, Yen-Ting Chiang, Wen-Nuan Cheng, Hsueh-Yu Li

**Affiliations:** 1 Department of Otorhinolaryngology-Head and Neck Surgery, Sleep Center, Linkou-Chang Gung Memorial Hospital, Taoyuan City 33305, Taiwan; 2 Department of Surgery, Linkou-Chang Gung Memorial Hospital, Taoyuan City 33305, Taiwan; 3 College of Medicine, Chang Gung University, Taoyuan City 33305, Taiwan; 4 Department of Pulmonary and Critical Care Medicine, Sleep Center, Linkou-Chang Gung Memorial Hospital, Taoyuan City 33305, Taiwan; 5 Department of Internal Medicine (Cardiology), Sleep Center, Taoyuan-Chang Gung Memorial Hospital, Taoyuan City 33305, Taiwan; 6 Department of Sports Sciences, University of Taipei, Taipei City 100, Taiwan

**Keywords:** obstructive sleep apnea, morbid obesity, bariatric surgery, combined airway and bariatric surgery (CABS)

## Abstract

Although continuous positive airway pressure is the gold standard for obstructive sleep apnea (OSA), it does not improve obesity. By contrast, bariatric surgery significantly improves obesity but with sustained OSA in the majority of patients. This study proposes a comprehensive technique—combined airway and bariatric surgery (CABS)—to improve both obesity and OSA. The aims of the study are to evaluate the feasibility, safety and efficacy of CABS in morbidly obese OSA patients. The retrospective study enrolled 20 morbidly obese OSA patients (13 males; median age, 44 years; median apnea–hypopnea index (AHI), 63.2 event/h; median body mass index (BMI), 41.4 kg/m^2^). The study population was divided into two groups based on patients’ volition after shared decision making. The bariatric surgery (BS) group included laparoscopic sleeve gastrectomy (LSG, *n* = 8) and laparoscopic Roux-en-Y gastric bypass (LRYGB, *n* = 2). CABS involved bariatric surgery—LSG (*n* = 8) and LRYGB (*n* = 2) in conjunction with airway surgery (concurrent nasal/palatal/lingual surgery [*n* = 8], concurrent palatal/lingual surgery [*n* = 2]). Patients received a home sleep apnea test at baseline and one year after surgery. The two patient groups were not different in age, sex, preoperative AHI or preoperative BMI. There was no airway compromise, wound bleeding or infection throughout the postoperative period. One year after surgery, patients in both groups achieved significant improvement in AHI and BMI. Furthermore, the improvement in AHI was significantly higher (*p* = 0.04) in the CABS group than in the BS group despite the insignificant change in BMI reduction (*p* = 0.63) between the two groups. In the CABS group, the BMI dropped from 40.4 to 28.4 kg/m2 (*p* < 0.0001) and the AHI decreased from 75.1 to 4.5 event/h (*p* = 0.0004). The classic success rate for OSA was 90% and the cure rate was 60% in the CABS group. A perioperative assessment of CABS group patients also revealed a significant improvement in the Epworth sleepiness scale, minimal O_2_ saturation, snoring index and heart rate. CABS is feasible and safe in treating OSA with morbid obesity. CABS achieves more reduction in AHI than BS alone with high success and cure rates for OSA. CABS can also significantly improve quality of life and general health and offers a comprehensive alternative for morbidly obese OSA patients.

## 1. Introduction

Obstructive sleep apnea (OSA) is defined as repeated episodes of obstructive apnea and hypopnea during sleep [1]. OSA is a chronic age-related disease that may lead to cardiovascular, neurovascular, neurocognitive, metabolic, pulmonary and psychiatric consequences and even fatality in traffic accidents [2]. The etiology of OSA seems to be multifactorial in that it involves anatomy, muscle tone, arousal and loop [3]. From the clinical viewpoint, the cause of OSA can be attributable to local hypertrophy of soft tissue, regional craniofacial anomaly and systemic obesity [4]. Among them, obesity plays an increasingly important role in the etiology because obesity has become a growing issue worldwide in recent decades [5,6]. 

The relationship between OSA and obesity can be bidirectional [7]. A ten-percent weight gain leads to a sixfold increase in the odds of developing OSA, which results in a greater prevalence of OSA in obese patients compared to the general population [8]. On the other hand, over half of adult OSA patients have obesity [9]. Another study found that the incidence of undiagnosed OSA in bariatric surgery patients, who are morbidly obese, varies from 35% to 94% [10]. Because the reciprocal causation between obesity and OSA is evident, morbidity and mortality rates increase if either of them is left untreated. 

**Figure 1:**
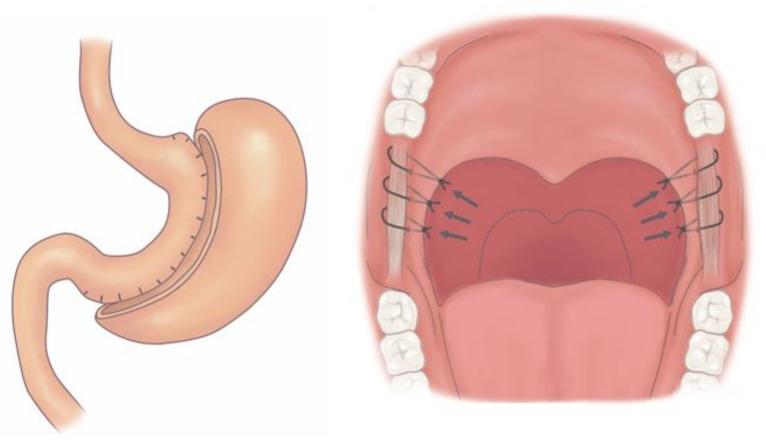
Combined airway and bariatric surgery (CABS) to solve obesity and obstructive sleep apnea at once.

OSA is present in almost two-thirds of morbidly obese patients [11]. Regarding treatment for morbidly obese OSA patients, continuous positive airway pressure (CPAP) is the first-line treatment. However, there is a large percentage of patients who are intolerant to CPAP therapy. Further, morbidly obese OSA patients even with good adherence to CPAP are still at a high risk of morbidity because of persistent obesity [12]. Bariatric surgery is an alternative to CPAP by reducing weight to decrease many co-morbidities including OSA [13]. Bariatric surgery (BS) for morbidly obese OSA patients is generally effective in reducing adverse respiratory events due to the widening of the airway by the volume reduction of adipose tissue. However, the efficacy can be suboptimal for sustained OSA in a large proportion of subjects because airway obstruction can be attributable, at least partially, to hypertrophic (lingual) tonsils, a redundant soft palate, deviated septal cartilage or weakness of pharyngeal muscle tone, [4] and those are beyond the scope of BS. One study revealed that AHI improved from 47.9 to 24.5 event/hour after BS in morbidly obese OSA patients. Additionally, the majority (71%) retained moderate-to-severe OSA postoperatively [14]. Another study showed a high residual AHI in patients after BS (44.9 vs. 29.2) and unveiled poor compliance with postoperative CPAP therapy [15]. Airway surgery, straightforwardly widening the obstructed airway, was considered a salvage treatment in OSA patients with a high residual AHI and clinical symptoms after BS. However, repeated intubation and extubation increase the risk of airway compromise throughout the perioperative periods in morbidly obese OSA patients [16]. Consequently, there is a need to combine airway and bariatric surgery to maximize surgical outcomes in morbidly obese OSA patients and solve the two co-morbidities at once. (Figure 1) The aims of this study are to investigate the feasibility, safety and effectiveness of the comprehensive technique of combined airway and bariatric surgery (CABS) in morbidly obese OSA patients. Results from this study may be helpful in contributing to the evolution of treatment guidelines for morbidly obese OSA patients.

## 2. Methods

### 2.1. Ethics Statement

This retrospective study was approved by the Institutional Review Board (IRB) of the Chang Gung Medical Foundation (number: 202200743B0) and by waivers of the patients’ informed consent. The Linkou Chang Gung Memorial Hospital is the main branch of the Chang Gung Memorial Hospital. The IRB of the Chang Gung Medical Foundation is the representative and in charge of all branches of the Chang Gung Memorial Hospital in IRB review affairs.

### 2.2. Study Population 

This single-center observational study was conducted between 2019 to 2020 at Chang Gung Memorial Hospital (Linkou). Eligible candidates were adults diagnosed with OSA (apnea–hypopnea index [AHI] > 5 event/hour) with OSA-related symptoms (snoring and daytime sleepiness). Patients were initially instructed to lose weight through lifestyle modification but failed. All patients were intolerant to CPAP trials and seeking surgical intervention. The inclusion criteria included age between 20 and 65 years, body mass index (BMI) > 32 kg/m^2^ with obesity-related comorbidities or BMI > 37 kg/m^2^ with or without comorbidities [17], AHI > 15/hour, complete questionnaire (Epworth sleepiness scale) [18] and complete data of the home sleep apnea test [19] at baseline and 12 months after surgery. The exclusion criteria included significant craniofacial anomaly (syndromic patient), combined severe medical disease and high risk for general anesthesia (American Society of Anesthesiologists physical status class > 2) [20]. The study population was divided into two groups based on patients’ willingness after shared decision making. 

### 2.3. Surgical Technique

BS included laparoscopic sleeve gastrectomy [21] (LSG) and laparoscopic Roux-en-Y gastric bypass [22] (LRYGB) for this study population. LSG was implemented as the first priority, and LRYGB was used in patients with either gastroesophageal reflux (Los Angles Grade B using a preoperative esophagogastroduodenal scope), hiatal hernias or poorly controlled diabetes. Patients were placed in a supine position, and four trocars were inserted (two 12 mm, one 11 mm and one 5 mm trocar). For LSG, dissection was started with the detachment of the greater omentum 4 cm from the pylorus up to the angle of His using a Harmonic Scalpel^®^ (Johnson & Johnson Medical KK, Ethicon Endo-Surgery, Cincinnati, OH). A Fr. 32 oral gastric tube was inserted along the lesser curvature to the gastric antrum as a guide. Sleeve formation was performed by resection of the greater curvature side of the stomach with an endoscopic linear cutter and reinforcing the staple line with seromuscular sutures. The steps of LRYGB were started by creating the gastric pouch below the first branch vessel of the lesser curvature side using a 45 mm endoscopic linear cutter and continuing upward to the angle of His. A small gastrojejunostomy was made with a 120 cm alimentary limb followed by a jejunojejunostomy with a 70 cm biliopancreatic limb. Both the mesenteric defect and Peterson space were closed with non-absorbable stitches. In both procedures, no drain tube was left. Patients were allowed to sip water immediately after surgery followed by a clear liquid diet on the first postoperative day and a pureed diet for the remaining two weeks. 

CABS was implemented with bariatric surgery by a general surgeon and airway surgery by an otorhinolaryngologist sequentially in the same operation. 

Airway surgery involved nasal surgery, palatal surgery and tongue surgery. 

**Figure 2:**
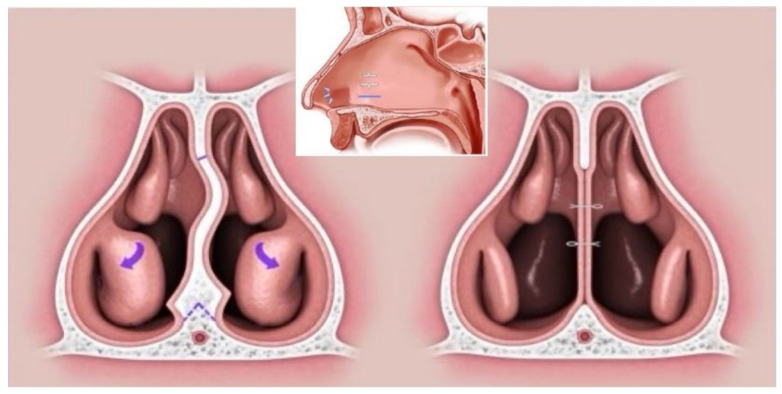
Mini-invasive septoturbinoplasty (MIST) involves excision of deviated septum, triangular hump preservation of nasal floor, incision drainage along nasal floor and trans-septal sutures and out-fracture of inferior turbinate. Arrow: out-fracture of inferior turbinate.

Nasal surgery was performed by mini-invasive septoturbinoplasty (MIST) that involved excision of the deviated septum, triangular hump preservation of the nasal floor to protect the great palatine artery, incision drainage along the nasal floor and trans-septal horizontal sutures to avoid septal hematoma and out-fracture of the inferior turbinate [4] (Figure 2).

**Figure 3:**
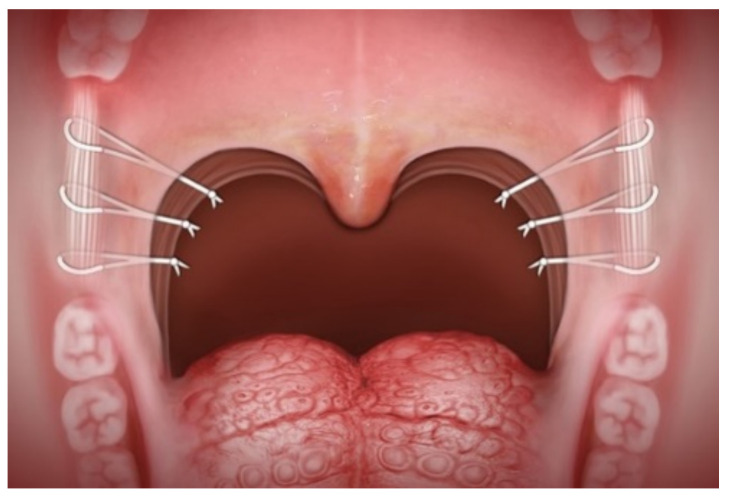
Raphe-based omni-suspension of the soft palate.

Palatal surgery was implemented through palatal hybrid surgery that involved pillar-mucosa-preserved tonsillectomy, ablation of supratonsillar adipose tissue, stereoscopic reconstruction of the tonsillar fossa (pharyngoplasty), raphe-based omni-suspension of the soft palate (palatoplasty) [23] (Figure 3) and advancement of the uvula (uvuloplasty). 

**Figure 4:**
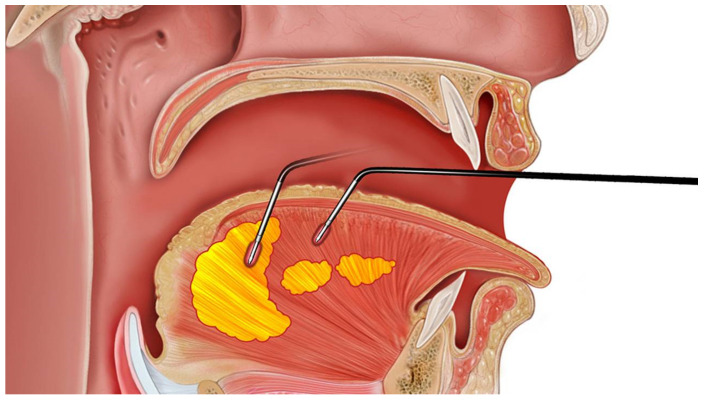
Coblation ablation tongue (CAT) involves multiple ablations of the tongue body muscle and tongue base fat with coblation wand.

For tongue surgery, coblation ablation tongue (CAT) involved the use of a coblation wand to perform multiple ablations of the tongue body muscle and tongue base fat [24] (Figure 4).

### 2.4. Home Sleep Apnea Test

Patients with sleep-related breathing disorders have received standard polysomnography in our sleep center for diagnosis before treatment for decades. However, our sleep center was repeatedly on lockdown due to the COVID-19 pandemic in the past two to three years. This largely impeded the normal schedule of in-lab sleep tests, and the fear of COVID-19 infection in the hospital further lowered the patients’ motivation to receive standard polysomnography. In this difficult situation, we decided to shift the in-lab sleep test to a home sleep test (HST) in part for the surgical sleep-disordered breathing patients in the interest of safety and the non-delayed diagnosis of OSA. For those patients co-morbid with insomnia, other sleep disorders or major medical diseases, standard polysomnography was arranged as usual. 

The HST used in this study was ApneaLink^®^ (ResMed, Sydney, Australia), which is an ambulatory sleep monitor that can detect sleep apnea with acceptable reliability 19. The parameters are measured and analyzed automatically and meanwhile reviewed and rescored by an experienced sleep specialist. A report was disused in the case of insufficient recording time (<4 h), chaotic signal or sensor loss, and the patient received repeated baseline HSTs. The outcome HST was arranged one year after CABS.

### 2.5. Statistical Analysis

Continuous variables were presented as the median (interquartile range). Differences between pre- and postoperative data were assessed using Wilcoxon Signed-rank tests. Differences in age, preoperative AHI, preoperative BMI and perioperative changes between the BS group and CABS group were assessed using Mann–Whitney U tests. Differences in sex between the two groups were assessed using Fisher’s Exact test. Correlations between two variables were assessed using Spearman’s Rank test. A two-sided *p* value < 0.05 was considered statistically significant. Statistical analyses were performed using SPSS for Mac 21.0 (Statistical Package for Social Sciences; SPSS Inc., Chicago, IL, USA).

## 3. Results

### Demographics

The study enrolled 20 morbidly obese OSA patients (13 males; median age, 44 years; median apnea–hypopnea index (AHI), 63.2 event/h; median body mass index (BMI), 41.4 kg/m^2^). There is no significant difference between the BS and CABS groups in age, sex, preoperative AHI, preoperative BMI or procedure for bariatric surgery. (Table 1) 

In the CABS group, physical examinations showed a high tongue position (modified Mallampati score III) in all patients and a variable tonsil size with a large tonsil size grade III/IV in 4 patients, a small tonsil size grade I/II in 6 patients; and a deviation of the nasal septum in 8 patients. 

The BS group included LSG (*n* = 8) and LRYGB (*n* = 2). CABS involved airway surgery (concurrent nasal/palatal/lingual surgery [*n* = 8], concurrent palatal/lingual surgery [*n* = 2]) in conjunction with BS using LSG (*n* = 8) or LRYGB (*n* = 2). 

All patients were intubated and extubated smoothly in the operating room. There was no airway compromise or wound bleeding/infection throughout the postoperative period. The hospitalization for the study population was 5 days. 

**Figure 5:**
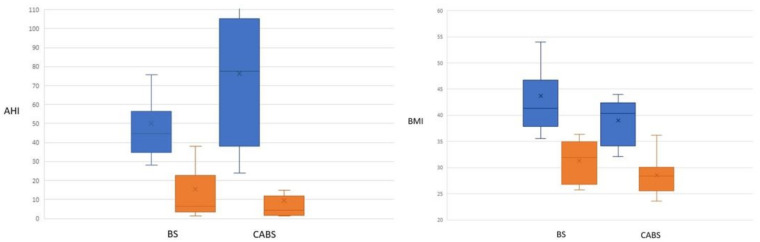
Perioperative changes in AHI and BMI in both BS group and CABS group. Preoperative (blue), postoperative (brown). CABS—combined airway and bariatric surgery, BS—bariatric surgery, BMI—body mass index, AHI—apnea–hypopnea index.

One year after surgery, patients in both groups achieved significant improvements (*p* = 0.005) in AHI (BS group: 44.7 vs. 6.5, CABS group: 75.1 vs. 4.5) and BMI (BS group: 41.3 vs. 31.9, CABS group: 40.4 vs. 28.4). (Figure 5) Despite insignificant perioperative changes in BMI reduction between the two groups (BS vs. CABS= 10.9 vs. 9.9 kg/m^2^, *p* = 0.63), perioperative improvement in AHI was significantly higher in the CABS group than in the BS group (65.7 vs. 31.5 event/hour, *p* = 0.04). 

**Figure 6:**
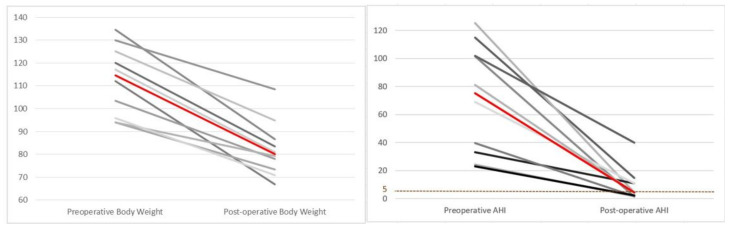
Perioperative changes in body weight and apnea–hypopnea index (AHI) in individual patients (CABS group). Red line: line of median change.

For the CABS group, reductions in individual body weight and AHI are demonstrated in Figure 6. 

**Figure 7:**
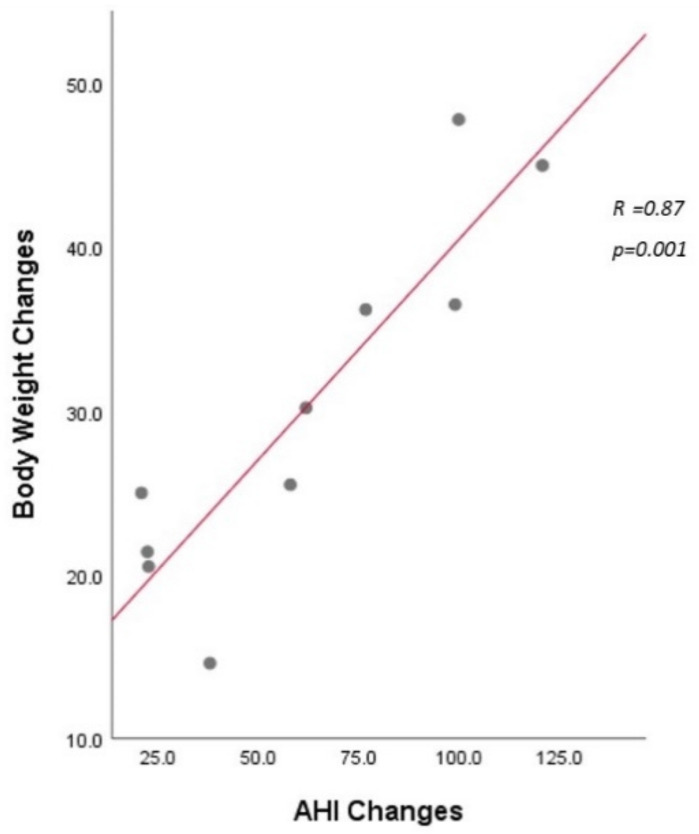
Changes in apnea–hypopnea index (AHI) are significantly correlated with changes in body weight (r = 0.87, *p* = 0.001) after combined airway and bariatric surgery.

The success rate for OSA in the CABS group was 90% (9/10) according to Sher’s criteria [25], and the cure rate (AHI < 5) was 60% (6/10), which was higher than the BS group with a success rate of 80% (8/10) and a cure rate of 50% (5/10). Besides, the apnea index, oxygen desaturation index, supine and non-supine AHI improved significantly, four saturation-related parameters including mean SpO2, minimal SpO2, <90% saturation also improved significantly after CABS. In addition, snore index and heart rate also decreased significantly by the one-year follow-up. Apart from objective changes in HSTs, CABS also significantly improved patients’ quality of life in terms of ESS (18 vs. 2, *p* = 0.004) and subjective snoring according to the visual analogue scale (10 vs. 1, *p* < 0.0001). (Table 2) Furthermore, the changes in AHI are significantly correlated with the changes in BMI (r = 0.83, *p* = 0.003) in the CABS group. By contrast, the changes in AHI are not significantly correlated with the changes in BMI (r = 0.49, *p* = 0.15) in the BS group. In addition, the changes in AHI are significantly correlated with the changes in body weight (r = 0.87, *p* = 0.001) in the CABS group (Figure 7).

## 4. Discussion

For morbidly obese OSA patients, BS resulted in a significant reduction in body weight, BMI and AHI. However, OSA persisted at follow-up in the majority of subjects. To the best of our knowledge, this is the first study using combined airway and bariatric surgery to improve surgical outcomes for morbidly obese OSA patients. The results show that CABS reduced AHI more than standalone BS with a success rate of 90% and a cure rate of 60%. Further, CABS significantly improved both quality of life and general health regarding daytime sleepiness, hypoxemia, snoring and elevated heart rate. 

The original intention of BS is the effective reduction of body weight. An update meta-analysis study showed that BS achieved significant reductions in mean body weight (133.2 kg vs. 95.6 kg, −35.4 kg, −26.6%) and mean BMI (47.8 vs. 34.5, −13.2 kg/m^2^, −27.6%) [26]. However, the pooled mean post-surgery weight (95.6 kg) and BMI (34.5 kg/m^2^) remained high and were still within the obese range. The study showed that CABS significantly reduced body weight (114.6 kg vs. 80.2 kg, −34.4 kg, −30.0%) and BMI (40.4 vs. 28.4, −12 kg/m^2^, −29.7%). The reduction in body weight and BMI was not different between the CABS and BS groups. This suggests that added airway surgery did not trigger further body weight reduction. The results of weight changes following BS are similar but with differences from previous studies, and that may be attributable to the different severity of obesity between Caucasian and Asian ethnic groups. 

A meta-analysis study of BS for OSA revealed that the pooled mean pre- and post-surgery AHI were 39.3 ± 15.1 event/h and 12.5 ± 5.6 event/h (overall *p* < 0.00001) with a change in the AHI of -25.1 event/h (64% reduction) [27]. One case series study (*n* = 110) assessing the effect of BS on OSA showed that the mean AHI significantly decreased from 39.5 to 15.6 event/h with the normalization of AHI (<5) in 25.5% [27]. Another recent comparative study on BS (*n* = 44) revealed that AHI improved from 44.9 to 29.2 event/h with a cure rate of 15.9%.15 A multicenter trial (*n* = 132) investigating the effect of BS on OSA revealed that AHI decreased from 27.8 to 9.9 event/h with a 45% cure rate, but moderate or severe OSA persisted in 20% of the patients after surgery [28]. One small sample size (*n* = 12) study revealed that AHI improved from 46.5 to 16 event/h and OSA was no longer present in 25% of the patients after BS [29]. These results suggest that BS can significantly reduce the severity of OSA but with a sustained high AHI and a variable and low success rate (depending on baseline AHI). The present study shows that CABS improved AHI from 71.5 event/h to 4.5 event/h (*p* = 0.0004) with a 93.7% reduction in baseline AHI and a 60% cure rate. In comparison with standalone BS, CABS had a higher reduction in baseline AHI. This difference may be attributable to two pathways: 1. The combined airway surgery in CABS reduced airway resistance by enlarging the airspace, which transforms into a decrease in intrathoracic negative pressure and consequently ameliorates airway collapse. 2. Nasal surgery in CABS reduces nasal resistance, which ameliorates oral breathing and makes the airway stable. It is well-known that OSA has a close interaction with sleep position [30]. Previous studies have revealed that weight loss improves non-supine AHI by a greater degree than supine AHI [31]. There was a distinct difference in the normalization of AHI according to weight loss between non-supine AHI and supine AHI (22% vs. 0%) [32]. In this study, both supine AHI (58.5 vs. 6.3, −89.2%) and non-supine (17.9 vs. 2.6, −85.5%) improved significantly after CABS. We assume that significant weight loss results in the reduction of fat in the lateral wall (parapharyngeal fat pat), which contributes to the reduction in non-supine AHI.32 By contrast, the decrease in supine AHI in our study can be attributable to procedures for airway surgery such as palatal suspension that lessens the anterior–posterior collapse of the soft palate, reconstruction of the tonsillar fossa that stabilizes the tongue and coblation ablation of the tongue that stretches the lingual muscle and ameliorates its anterior–posterior collapse during sleep. Furthermore, weight loss further reduces tongue fat volume, which mediates the reduction in AHI in obese patients with OSA [33]. In addition, we found that central sleep apnea also decreased from 4.3 to 0.6 event/h in one patient, which may be attributable to the improvement in respiratory drive due to a significant body weight reduction after BS [34]. These results suggest that CABS may achieve two-way efficacy in both positional and central sleep apnea, which warrants further research. 

It is noteworthy that the changes in AHI are significantly correlated with the changes in body weight (r = 0.87, *p* = 0.001) and the changes in BMI (r = 0.83, *p* = 0.003) following CABS. This is inconsistent with two previous randomized control trials addressing the effect of BS on OSA that demonstrated no relationship between the amount of weight loss and the improvement in OSA [35,36]. We speculate that BS alone did not cope with the anatomy-related OSA that came into being as a confounding factor. By contrast, CABS eliminates the role of anatomy-related OSA and, consequently, the amount of body loss can be associated with a decrease in the severity of OSA. 

Intermittent hypoxia is of paramount importance in contributing to major adverse cardiac events, and hypoxia-induced inflammation further augments these adverse effects in OSA patients [37,38]. Blood oxygen saturation (SpO_2_)-derived parameters including mean SpO_2_, lowest SpO_2_, <90% O_2_ saturation and oxygen desaturation index have been used to assess the severity of hypoxemia in OSA patients [39]. In this study, CABS significantly improved all hypoxemia-related parameters (mean SpO_2_, lowest SpO_2_, <90% O_2_, ODI) in morbidly obese OSA patients. These results indicate that CABS corrects patients’ hypoxemic essence in a multidimensional assessment. The improvement in hypoxemia also suggests that CABS ameliorates the systemic inflammation status of morbidly obese OSA patients. An elevated heart rate is associated with post-event tachycardia in OSA patients [40]. During one event of obstructive apnea/hypopnea with subsequent arousal and hypoventilation, a decrease in HR with a subsequent increase in HR is observed in OSA patients [41]. A previous study revealed that the severity of OSA is independently associated with the mean heart rate during sleep [42]. This suggests that the increased severity of OSA increases heart rate during sleep. The elevated mean heart rate in OSA patients could be attributable to: episodes of apnea/hypopnea increasing sympathetic nervous activation, hypoxia and hypercapnia further augmenting its activity and arousal immediately after hypopnea/apnea events inducing additional surges in the sympathetic neural outflow [43,44,45]. Additionally, research has demonstrated a positive association between elevated heart rate and adverse cardiovascular events in both the general population and cardiovascular disease patients [46,47]. Furthermore, the elevated heart rates in OSA patients can be significantly reduced following CPAP therapy [42,48]. Our data shows that CABS significantly reduces median heart rate (77 vs. 62, *p* < 0.0001) and maximal heart rate (114.0 vs. 94.3, *p* = 0.005) by the one-year follow-up. It is assumed that CABS lessens respiratory effort and consequently restores sympathetic nervous system overactivation during sleep, which potentially reduces the risk of future cardiovascular events in OSA patients. Dalmar et al. reported that patients with OSA, despite a similar initial weight loss after LAGB, had a higher incidence of cardiovascular events compared with a propensity-matched group without OSA, and treatment with CPAP appears to reduce such events [49]. These results emphasize the independent factor of OSA in cardiovascular events for obese patients and rationalize the necessity to treat both obesity and OSA together in obese OSA patients. Snoring is the most common complaint in OSA patients seeking treatment. Besides the effects from the combination with apnea/hypopnea, snoring alone can play a role as phonotrauma to damage adjacent organs such as the common carotid artery and hearing apparatus [49,50,51,52]. CABS significantly improves the snoring index from 479.1 to 20 event/h. The substantial amelioration of snoring after CABS is owing to the synergetic response due to the decreased velocity of airflow from the widening of the airway and decreased negative thoracic pressure from the decrement of abdominal fat. This tremendous decrease in snoring events suggests the vanishing of noisy breathing sounds and their phonotrauma effect in these patients. Daytime sleepiness is the principal symptom of OSA. CABS remarkably improves daytime sleepiness in terms of ESS (16.1 vs. 3.1, −13), and that is superior to the pooled data (11.1 vs. 5.6, −5.5) in a previous meta-analysis on bariatric surgery for morbidly obese OSA patients [26]. This difference could be attributed to the more patent and stable airway resulting from CABS, which reduces respiration-related arousal and fragmented sleep. 

There are several limitations of this study: 1. This is a small sample size study focused on morbidly obese OSA patients and, therefore, the findings of this study cannot be generalized to all OSA patients. More studies with big sample sizes are needed. However, this study is pioneering and can be used in patients with specific characteristics. 2. The use of home sleep testing may underestimate the severity of OSA compared to standard polysomnography. However, we excluded sleep-disordered breathing patients co-morbid with insomnia and other sleep disorders to lessen the potential bias of underestimation. Further, the data were reviewed and rescored by an experienced sleep specialist to correct the faulty signals before the report was dispatched. 3. This study is a retrospective case-controlled study. A randomized controlled trial would definitely provide more high-level evidenced outcomes, if available. The one-year outcomes of CABS may not ensure its efficacy in the long term, because the surgical effects on OSA may decline with time, not to mention the possible regaining of body weight. As a result, these patients will be followed regularly for possible weight gain or deterioration in OSA and to see whether salvage treatment is needed.

## 5. Conclusions

This study proposes a new combined surgery, CABS, to treat both OSA and obesity-related manifestations simultaneously. The low morbidity after CABS reflects its safety and feasibility. The high success and cure rate for OSA indicate CABS’s justifiability in treating this extremely tricky group of OSA patients. CABS offers a comprehensive alternative for respiratory and metabolic treatment as holistic care for morbidly obese OSA patients.

## Figures and Tables

**Table 1:** Baseline anthropometric, polysomnographic and surgical data.

Variable	CABS (*n* = 10)	BS (*n* = 10)	*p* Value
Age (year)	44 (34–47)	36.5 (29–46.3)	0.28
Gender (Male/female)	7/3	6/4	1
BMI (Kg/m^2^)	40.4 (34.1–42.4)	41.3 (37.9–46.7)	0.25
AHI (event/hour)	75.1 (30.9–105.1)	44.7 (34.7–56.4)	0.19
Bariatric surgery (LSG/LRYGB)	8/2	8/2	1

Data are expressed as median (interquartile) or ratio; Abbreviations: CABS—combined airway and bariatric surgery, BS—bariatric surgery, BMI—body mass index, AHI—apnea–hypopnea index, LSG—laparoscopic sleeve gastrectomy, LRYGB—laparoscopic Roux-en-Y gastric bypass.

**Table 2:** Changes in polysomnographic and anthropometric data after CABS.

Variables	Preoperative Median (Interquartile)	Postoperative Median (Interquartile)	*p* Value
Snore (VAS)	10 (9–10)	1 (0–2)	<0.0001
ESS	18 (13–20)	2 (0–6)	0.004
AHI (event/hour)	75.1 (30.9–105.1)	4.5 (1.8–13.5)	0.0004
Supine AHI	58.5 (24.1–104.9)	6.3 (2.2–33.3)	0.003
Non-supine AHI	17.9 (0–97.7)	2.6 (1.4–6.3)	0.02
AI (event/hour)	52.8 (3.8–84.1)	1.1 (0.2–3.4)	0.009
Mean SAT (%)	87.0 (78.3–94.0)	94.0 (93.8–95.3)	0.008
Mini SAT (%)	58.5 (40.8–77.3)	87 (75.5–90.0)	0.001
ODI (event/hour)	66.4 (31.2–99.2)	5 (2.3–12.9)	0.0003
<90% SAT (%)	30 (4.5–75.5)	1 (0–9.3)	0.006
SI (event/hour)	479.1 (355.5–592)	20 (6.9–168.7)	0.0001
HR (beat/minute)	77 (72–86)	62 (55–66)	<0.0001
Max HR (beat/min)	114.0 (110–181)	94.3 (88.3–100)	0.0005
Body weight (kg)	114.6 (95.5–127.5)	80.2 (72.9–90.8)	<0.0001
BMI (kg/m^2^)	40.4 (34.1–42.4)	28.4 (25.6–30.1)	<0.0001

Abbreviations: CABS—combined airway and bariatric surgery; ESS—Epworth sleepiness scale; AHI—apnea–hypopnea index; AI—apnea index; Mini SAT—minimal O_2_ saturation; ODI—oxygen desaturation index, SI—snore index, HR—heart rate, Max HR—maximal heart rate, BMI—body mass index.

## Data Availability

The data are not publicly available due to the regulation of our institution and protection of patients’ privacy particular in small sample size group. However, the data presented in this study are available on request from the corresponding author for further research, if available.
